# 2519: A quantitative disintegration method to evaluate polymeric films

**DOI:** 10.1017/cts.2017.238

**Published:** 2018-05-10

**Authors:** Sheila Grab, Yvonne Cosgrove Sweeney, Dorothy L. Patton, Lisa C. Rohan

**Affiliations:** University of Pittsburgh, Pittsburgh, PA, USA

## Abstract

OBJECTIVES/SPECIFIC AIMS: To establish an in vitro quantitative method for the evaluation of polymeric film disintegration that can be applied to predict in vivo behavior. METHODS/STUDY POPULATION: Two clinically advanced vaginal microbicide film products containing tenofovir and dapivirine were used as model films throughout this work. Films were made using the solvent cast manufacturing method in which polymers, excipients, plasticizer, and APIs were either dissolved or dispersed in water, mixed, and cast on a heated substrate. The novel, quantitative method was developed using a TA.XT Plus Texture Analyzer^®^ (Texture Technologies) in combination with a TA-108S5 fixture and the TA-8A: 1/8″ diameter rounded end ball probe. Exponent^®^ was used as the data analysis software. In this method, the film was placed and secured in the fixture, the probe applied a constant force to the film product, and a biologically relevant amount of fluid was applied to the film. The probe was able to penetrate the film upon disintegration resulting in an applied force of zero at that point. A curve of force Versus time was plotted, and disintegration time was defined as the time between fluid addition until the probe force reached zero. Test parameters were optimized in order to reduce error. Visual observation of film disintegration was conducted in the in vivo macaque model using films that included a water-soluble blue dye for film visualization. Colpophotography was also used to confirm film disintegration. In vitro results were compared with in vivo findings. RESULTS/ANTICIPATED RESULTS: The Texture Analyzer disintegration method developed provided quantitative disintegration times and did not rely on user defined endpoints which is common in many visual disintegration tests. The disintegration method was able to distinguish differences between the 2 clinical film products and produced reproducible disintegration times for the tenofovir and dapivirine films. The tenofovir film had a shorter disintegration time (41.28±2.85 s) compared with that of the dapivirine film (88.3 6±9.82 s). This method was also able to distinguish changes made to these 2 clinical film products in terms of volume and formulation alterations. In vitro and in vivo disintegration times differed by orders of magnitude, with in vitro time being measured in seconds and in vivo time being measured in days, for a variety of factors, mainly the application of constant force to the film product. Regardless of these differences, the rank order of film disintegration remained constant for in vitro and in vivo disintegration and an In Vitro In Vivo Correlation (IVIVC) trend could be seen. DISCUSSION/SIGNIFICANCE OF IMPACT: Standardization of preclinical in vitro assessments which minimize user bias are crucial to the field of pharmaceutical film development. As this field continues to develop and more products advance for pharmaceutical application, this method has the potential to become a standard assessment of film functionality. This study represents a first step in the process of developing an IVIVC. More films will need to be tested using both in vitro and visual methods in order to establish and accurate factor to predict in vivo behavior.

